# Lifetime Measurements in ^178^Hf

**DOI:** 10.6028/jres.105.016

**Published:** 2000-02-01

**Authors:** R. C. de Haan, A. Aprahamian, H. G. Börner, C. Doll, M. Jentschel, A. M. Bruce, S. R. Lesher

**Affiliations:** Physics Department, University of Notre Dame, Notre Dame, Indiana 46556; Institut Laue-Langevin, 38042 Grenoble, France; University of Brighton, Brighton, BN2 4GJ, United Kingdom; Physics Department, University of Notre Dame, Notre Dame, Indiana 46556; Indiana University South Bend, South Bend, IN 46616

**Keywords:** level lifetimes, phonon excitations, radioactive hafnium

## Abstract

Lifetimes of levels from K^π^ = 2^+^, K^π^ = 4^+^ and several K^π^ = 0^+^ bands have been measured in the ^178^Hf nucleus using the GRID technique. Lifetimes of the 2^+^ and 3^+^ levels were measured within the K^π^ = 2^+^ *γ* band. A lower limit was established for the lifetime of the 4^+^ level of the K^π^ = 4^+^ band. The resulting upper limits for the absolute *B*(E2) values exclude collective transitions from the K^π^ = 4^+^ to the ground state band but not to the K^π^= 2^+^ band. Level lifetimes were also measured for several states within three separate K^π^= 0^+^ bands. Evidence is presented for a previously unobserved case of two excited K^π^= 0^+^ bands being connected via collective E2 transitions.

## 1. Introduction

Collective phonon excitations are common to the descriptions of vibrational motion in various many-body systems including nuclei, atoms, molecules, and metal clusters. In nuclei, the lowest order shape oscillations are quadrupole in nature resulting in two types of vibrational excitations for deformed nuclei; *β* with no angular momentum projection on the symmetry axis and *γ* with a projection of K = 2^+^. Two-phonon excitations can in principle be constructed from one or more quanta of these *β* and *γ* vibrations to form *ββ*, *βγ*, and *γγ* types of vibrations [1].

Single phonon vibrational excitations are abundant in the nuclear landscape while the existence of two-phonon vibrational excitations continues to be the focus of a lively debate in nuclear structure studies. A recent study by Wu et al. [2] presented a compilation of the excitation energy ratios of possible two-phonon *γγ*(K = 4^+^) to one-phonon *γ*(K = 2^+^) bandhead levels for all deformed nuclei in the rare-earth region of the chart of nuclides. The compilation was based on the identification of K = 4^+^ bands with a strong preference in decay to the K = 2^+^*γ* band. The energy ratios showed a variation in value from 1.29 in ^178^Hf to 2.89 for the ^164^Dy nucleus. Preference of decay however is not by itself a signature of two-phonon character. It is necessary to know the absolute E2 transition probabilities connecting the various excitations. The first measurement of lifetimes to extract absolute *B*(E2) values resulted in the observation [3] of a two-phonon *γγ* (K = 4^+^) band in ^168^Er where the energy ratio of two to one phonon excitations is 2.50. Recently, there have been several observations of two-phonon *γγ*(both K = 4^+^ and K = 0^+^) vibrational excitations [2–8]. In all of these nuclei the energy ratio of two-phonon to one-phonon bandheads is greater than 2.5. The lowest ratio in the compilation of Ref. 2 is a value of 1.29 for the ^178^Hf nucleus showing a K = 4^+^ band at 1513.828 keV with a preference of decay to the gamma band at 1174.626 keV. Using the GRID technique [9–11] we have undertaken a detailed study of level lifetimes in this nucleus in order to measure absolute *B*(E2) values. Level lifetimes measured were from several bands including the first excited K = 2^+^ and K = 4^+^ bands, as well as several K = 0^+^ bands. ^178^Hf is quite well known due to a number of extensive studies including (n, *γ*) [12] and (*α*,xn)reactions [13], ARC measurements, conversion electron studies [12], (d,d’) [14,15], (d,p) [14,15], (d,t) [14,15], (p,t) [16] and (p,*α*) [17] transfer reactions, and beta decay.

## 2. Experimental Procedure

The GRID technique allows lifetime measurements of levels populated in thermal neutron capture by measuring the Doppler broadening of depopulating transitions. The Doppler broadening is caused by previously emitted *γ*-rays giving the nucleus isotropically distributed recoil velocities. The recoil velocities are very small (typically 10^−4^ *c* to 10^−6^ *c*) with resulting broadening effect on the order of a few eV and very short slowing-down times in the target. The last point limits the optimum range of accessible lifetimes to a few picoseconds and lower. The line shape of a particular transition is measured using a double flat crystal spectrometer (GAMS4) installed 15 m from the core of the high flux reactor of the Institute Laue Langevin in Grenoble, France. The target consisted of 9.592 g of natural Hf oxide. The line shapes or specifically the wavelengths of chosen *γ* rays are measured by Bragg diffraction on ideal crystals where the energy resolution may be as good as Δ*E*/*E* ≈2 ×10^−6^. The broadened line shapes were fit with the GRIDDLE [18] code.

The input parameters of the program GRIDDLE include recoil velocity, temperature of the target, and the response function of the instrument in order to calculate the lifetime. The recoil velocity distribution is a function of the feeding of the level of interest. In cases where the feeding of a particular nuclear level is not completely known, we have made rather extreme assumptions for the missing feeding in order to extract conservative *upper* and *lower* limits. The *upper* limit of the extracted lifetime is determined by attributing the missing feeding intensity to a cascade of *γ*-ray transitions from the compound capture state. The *lower* limit is extracted by assuming that the missing feeding comes from the unplaced low energy transitions that were measured in this nucleus. The more realistic scenario would probably lie somewhere in the middle of the lifetimes resulting from these intentionally extreme feeding assumptions.

## 3. Results and Discussion

The measured lifetimes and the extracted *B*(E2) values are tabulated in Table 1. Table 2 shows a comparison of the experimental *B*(E2) values with the Alaga rules. The experimental *B*(E2) values have been normalized within individual depopulating states and bands.

### 3.1 K = 2^+^ Band

The 2^+^ bandhead of the *γ* band is at an excitation energy of 1174.626 keV. The lifetime of this level was previously known and was remeasured here as a test. The measurement yields a lifetime range of 0.27 ps to 1.27 ps resulting in a *B*(E2:2^+^ → 0^+^) range of 2.7 W.u. to 12.8 W.u. which is in agreement with a previous Coulomb excitation lifetime measurement [19] of 0.90 ± 0.03 ps and $B\left(E2:2_{\gamma }^{+}\to 0_{g.s.}^{+}\right)$ value of (3.9 ± 0.5) W.u. [20]. The 3^+^ level of this band is at an excitation energy of 1268.536 keV. This is the first measurement of a lifetime for this state yielding a range of 0.51 ps to 2.32 ps. For the 2^+^ and 3^+^ levels, 46 % and 37 % of the feeding is known, respectively.

### 3.2 K = 4^+^ Band

**Fig. 1:**
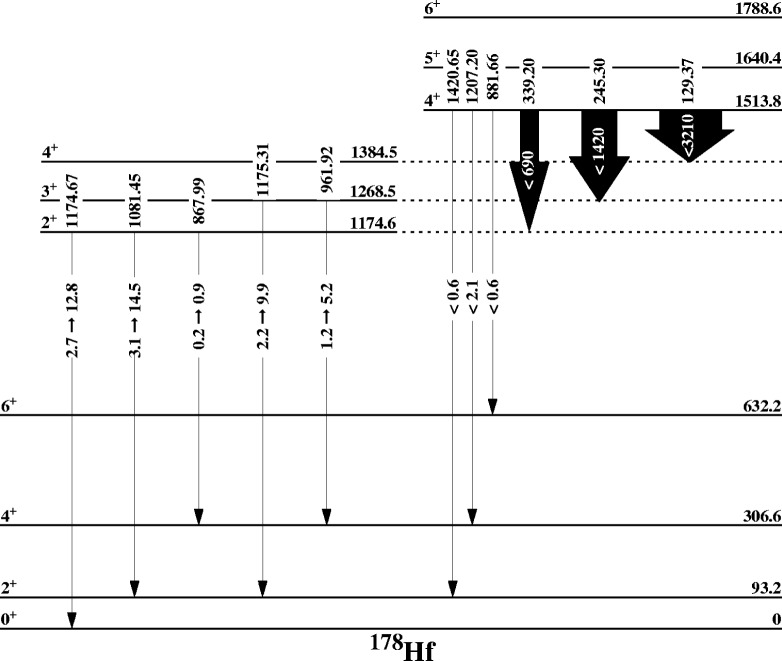
All the known K^π^ = 0^+^ bands in the nucleus 178Hf.

The 4^+^ bandhead level of the K = 4^+^ band is at an excitation energy of 1513.828 keV. 58 % of the feeding of this level is known. The line shape of the 1207.204 keV transition depopulating the 4^+^ level was measured in 12 separate scans. The result was that we were only able to determine a lower limit for the lifetime of *τ* ≥ 0.9 ps. We were unable to extract a meaningful higher limit since the convergence of the GRIDDLE code gave a lifetime longer than the slowing down time of the Hf nuclei indicating a longer lifetime for the level. This lower limit (upper limit in *B*(E2)) is by itself quite informative. The transitions to the g.s. band yield $B\left(E2:4_{K=4^{+}}^{+}\to J_{g.s}^{+}\right)$ values of less than 0.6 W.u. and 2.1 W.u. while the $B\left(E2:4_{K=4^{+}}^{+}\to J_{{K=2}^{+}}^{+}\right)$ values do not exclude a high degree of collectivity for the transitions connecting the K = 4^+^ and the K^π^ = 2^+^ bands. Figure 1 shows a partial level scheme of the K^π^= 2^+^ and K^π^= 4^+^ bands and their depopulating transitions in W.u. The single phonon $B\left(E2:2_{K=2^{+}}^{+}\to 0_{g.s.}^{+}\right)$ is measured to be approximately 4 W.u., the expected harmonic collective strength for two-phonon excitations is 2.78 times the collectivity of the single phonon. If this K^π^ = 4^+^ band is a two-phonon *γγ* vibrational excitation then the expected level lifetime should be on the order of 70 ps. Further comparisons can be made between the J →J transitions depopulating this 4^+^ level since these transitions are not affected by mixing matrix elements. For example, the $B\left(E2:4_{K=4^{+}}^{+}\to 4_{K=2^{+}}^{+}\right)$ in comparison with the $B\left(E2:4_{K=4^{+}}^{+}\to 4_{g.s.}^{+}\right)$ yield limits of < 1420 and < 2, respectively. The ΔK = 4 transitions are not allowed by the Alaga rules.

### 3.3 K = 0^+^ Bands

**Fig. 2:**
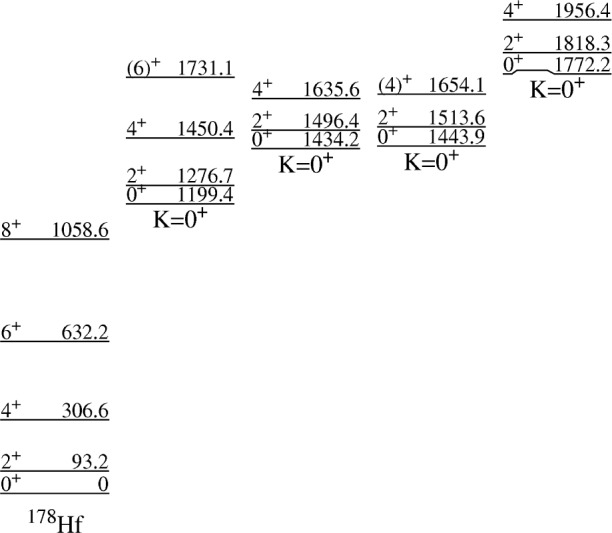
A portion of the ^178^Hf level scheme showing the *B*(E2) values extracted from the measured lifetimes shown in Table 1. *B*(E2) values are calculated from the E2 component of the transition intensity.

Figure 2 shows all the known K^π^ = 0^+^ bands below 2 MeV in the spectrum of the ^178^Hf nucleus. The specific K^π^ = 0^+^ bands are distinguished by a subscript referring to the order of excitation energies for the five bands. For example, the band at 1772.2 keV is labelled as the $K^{\pi }=0_{5}^{+}$ band. The first excited K^π^ = 0^+^ band is at 1199.4 keV. The lifetime of the 0^+^ bandhead level is not known. The 2^+^ member of this band at 1276.7 keV had a previously measured lifetime of (8.8 ± 3.5) ps determined by Coulomb excitation [19,20] resulting in $B\left(E2:2_{K^{\pi }=0_{2}^{+}}^{+}\to 0_{g.s.}^{+}\right)$ and $B\left(E2:2_{K^{\pi }=0_{2}^{+}}^{+}\to 4_{g.s.}^{+}\right)$ values of 0.06 W.u. and 0.38 W.u., respectively. This measurement with relatively large error bars had led to the conclusion that the first excited K^π^ = 0^+^ band is not collective. Here we report on a new measurement of the 4^+^ state lifetime of the same band at 1450.363 keV. The resulting $B\left(E2:4_{K^{\pi }=0_{2}^{+}}^{+}\to 6_{g.s.}^{+}\right)$ value range is 1 W.u. to 17 W.u. typical for transitions between a single-phonon vibrational excitation and the ground state band. The extracted *B*(E2) values depopulating this level indicate that the 4^+^ state at 1450.4 keV is a member of this K^π^ = 0^+^ band since it is connected to the 2^+^ member of the band by a collective 173.7 keV transition [20,21]. The $B\left(E2:4_{K^{\pi }=0_{2}^{+}}^{+}\to 6_{g.s.}^{+}\right)$ value falls within the range of expected collectivity of *β* vibrational excitations in contrast to previous classifications of this band as a quasi-particle excitation.

The next two K^π^ = 0^+^ bands in this nucleus are at excitation energies of 1434.239 and 1443.939 keV. The lifetime of the 1496.454 keV level (the 2^+^ member of the $K^{\pi }=0_{3}^{+}$ 3 band at 1434.239 keV) was measured while levels of the 1443.9 keV band were not due to the weak intensities of the depopulating transitions. The resulting range of *B*(E2) values also indicate degrees of collectivity typically observed from single-phonon *β* vibrational excitations.

**Fig. 3:**
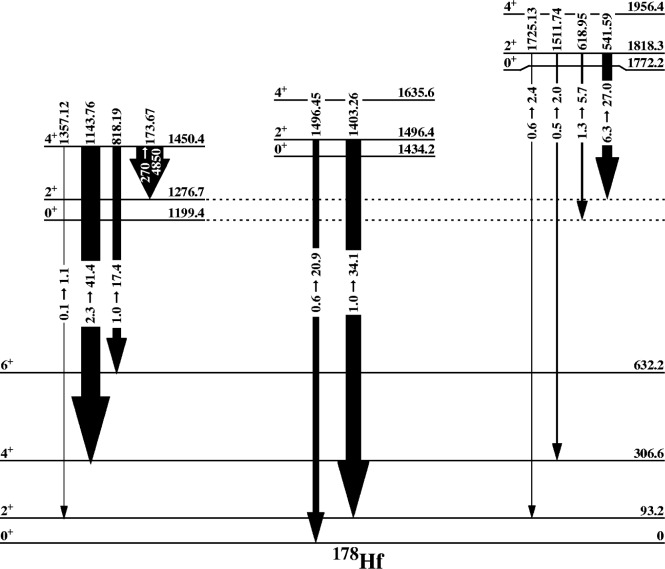
Partial level scheme showing the *B*(E2) values depopulating the K^π^ = 2^+^ and the K^π^ = 4^+^ bands.

A very interesting result concerns the $K^{\pi }=0_{5}^{+}$band in ^178^Hf at an excitation energy of 1772.2 keV. The extracted $B\left(E2:2_{K^{\pi }=0_{5}^{+}}^{+}\to 0_{K^{\pi }=0_{2}^{+}}^{+}\right)$ and $B\left(E2:2_{K^{\pi }=0_{5}^{+}}^{+}\to 0_{K^{\pi }=2_{2}^{+}}^{+}\right)$ are strong and highly collective. The 618.954 keV and 541.593 keV transitions depopulate the 2^+^ level of the $K^{\pi }=0_{5}^{+}$ band to the 0^+^ and 2^+^ members of the first excited $K^{\pi }=0_{2}^{+}$ band at 1199.380 keV and 1276.691 keV, respectively. The multipolarity of the 618.9 keV transition is E2 while the 541.6 keV transition is M1 + (50 ± 11)% E2 [12]. The deduced $B\left(E2:2_{K^{\pi }=0_{5}^{+}}^{+}\to 0_{K^{\pi }=0_{2}^{+}}^{+}\right)$ has a range of 1.3 W.u. to 5.7 W.u. while the $B\left(E2:2_{K^{\pi }=0_{5}^{+}}^{+}\to 0_{K^{\pi }=0_{2}^{+}}^{+}\right)$ range is 6.3 W.u. to 27 W.u for the *E*2 component of the transition. The observed high level of collectivity for the *E*2 component of the 541.59 keV transition is of particular importance since it is a J → J transition and therefore not affected by mixing [1,22] matrix elements. Figure 3 shows the depopulating *B*(E2) values from all three K^π^ = 0^+^ bands.

This new observation of two excited K^π^ = 0^+^ bands connected by collective transitions is the first case of its type. The observed preference of decay of the $K^{\pi }=0_{5}^{+}$ band at 1772.2 keV band to the $K^{\pi }=0_{2}^{+}$ 2 band at 1199.4 keV is compatible with the expected behavior of a collective vibrational excitation built on the 1199.4 keV band.

**Fig. 4:**
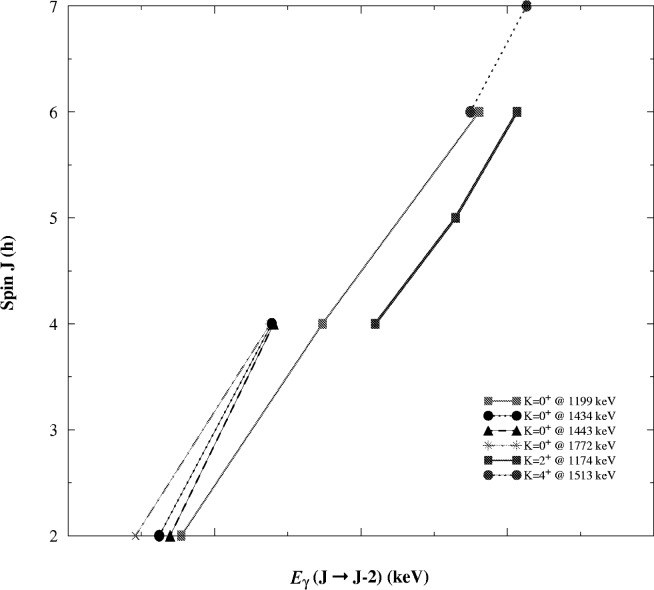
Dynamic moments of inertia for the K^π^ = 0^+^, K^π^ = 2^+^ and the K^π^ = 4^+^ bands in ^178^Hf.

One piece of evidence which further supports the relationship of these two K^π^ = 0^+^ bands at 1199.4 keV and 1772.2 keV is their identical dynamic moments of inertia. It had previously been shown that single and double gamma vibrational excitations exhibit identical dynamic moments of inertia [2]. Figure 4 shows the dynamic moments of inertia for the K^π^ = 0^+^, K^π^ = 2^+^, and K^π^ = 4^+^ bands. Dynamic moments of inertia for the K^π^ = 0^+^ bands at 1199.4 keV and 1772.2 keV are identical while the other two K^π^ = 0^+^ bands at 1434.2 keV and 1443.9 keV show moments of inertia which are very similar to each other but quite different from the g.s., the $K^{\pi }=0_{2}^{+}$, and the $K^{\pi }=0_{5}^{+}$ bands.

### 3.4 Conclusions

Level lifetimes have been measured in a K^π^ = 2^+^ band at 1174.626 keV, a K^π^ = 4^+^ at 1513.828 keV, and three excited K^π^ = 0^+^ bands in the ^178^Hf nucleus using the GRID technique. The measurement for the 2^+^ member of the K^π^ = 2^+^ band agrees with a previous coulex measurement. In addition we have measured the lifetime of the 3^+^ member of the same band. The results for the 4^+^ member of the K^π^ = 4^+^ band point to a lower limit in lifetime but no upper limit could be extracted. The measurement of the lifetime for the 2^+^ member of the $K^{\pi }=0_{3}^{+}$ band does not exclude this band from being characterized as a single-phonon *β* vibrational excitation. However, the strong collective transitions between the $K^{\pi }=0_{5}^{+}$ band at 1772.2 keV to the $K^{\pi }=0_{2}^{+}$ band at 1199.4 keV clearly point to a favoring of the $K^{\pi }=0_{2}^{+}$ band over the $K^{\pi }=0_{3}^{+}$. The $K^{\pi }=0_{5}^{+}$ band at 1772.2 keV is most likely a collective excitation built on the lower lying $K^{\pi }=0_{2}^{+}$ band at 1199.4 keV. This observation is very interesting and worth further investigation experimentally and theoretically.

## Figures and Tables

**Table 1:** Measured lifetimes for each level of interest along with extracted *B*(E2) values^a^

*E_x_*(keV)	*τ*(ps)	*E_γ_*(keV)	*I_γ_*	*α*	Multipolarity	*B*(E2) W.u.
1174.63	0.27 < *τ* < 1.27^b^	1174.67	11.167	0.00206	E2	2.73→12.78
		1081.45	8.401	0.00317	E2	3.10→14.53
		867.99	0.165	0.0037	E2	0.18→0.85
1268.54	0.51 < *τ* < 2.32	1175.31	13.124	0.00230	E2	2.20→9.94
		961.92	2.513	0.00424	E2	1.15→5.18
1450.36	0.14 < *τ* < 2.60	1357.12	0.304	0.002	E2	0.06→1.12
		1143.76	4.759	0.0095	E0+(M1 and/or E2)	2.30→41.38^c^
		818.19	0.376	0.0045	E2	0.97→17.45
		173.67	0.045	0.50^d^	E2	270→4850
1496.45	0.03 < *τ* < 1.09^e^	1496.45	1.447	0.0017	E2	0.58→20.86
		1403.26	1.714	0.00929	E0+(M1 and/or E2)	0.95→34.05^c^
1513.83	0.94 < *τ*	1420.65	5.182	0.0021	E2	< 0.58
		1207.20	9.993	0.00293	E2(88±10 %)	< 2.06
		881.66	0.466	0.0026	E2	< 0.57
		339.30	4.778	0.0462	E2	< 690
		245.30	1.954	0.1358	E2	< 1420
		129.37	0.171	0.461	E2	< 3210
1818.28	0.24 < *τ* < 1.04	1725.13	1.440	0.00555	E0+(M1 and/or E2)	0.57→2.44^c^
		1511.74	0.593	0.00142	E2	0.46→1.95
		618.95	0.020	0.0013^d^	E2	1.33→5.68
		541.59	0.097	0.023	E2(50±11 %)	6.32→26.95

a Transition intensities and conversion coefficients from Haque et al. [12].

b The result of a previous measurement by Coulomb excitation [20,19] was (0.90±0.03) ps.

c No information is available on the E2 component of the transition; *B*(E2) value has been calculated as an upper limit assuming 100 % E2.

d Theoretical conversion coefficients were used in the absence of measured values [23].

e The result of a previous measurement by Coulomb excitation [20,19] was (1.3±0.3) ps.

**Table 2:** Normalized absolute *B*(E2) values in comparison with CG^2^. The experimental numbers have been normalized to one of the transitions depopulating the state.

*E_x_*(keV)	*τ*(ps)	${K,J}_{\text{initial}}^{\pi }$	*E_γ_*(keV)	${K,J}_{\text{final}}^{\pi }$	Multipolarity	*B*(E2)_exp_	CG^2^
1174.63	0.27 < *τ* < 1.27^a^	2,2^+^	1174.76	0,0^+^	E2	1.00	1.00
			1081.45	0,2^+^	E2	1.13	1.43
			867.99	0,4^+^	E2	0.07	0.08
1268.54	0.51 < *τ* < 2.32	2,3^+^	1175.31	0,2^+^	E2	1.00	1.00
			961.92	0,4^+^	E2	0.52	0.40
1450.36	0.14 < *τ* < 2.60	0_2_,4^+^	1357.12	0,2^+^	E2	1.00	1.00
			1143.76	0,4^+^	E0+(M1 and/or E2)	37^b^	0.91
			818.19	0,6^+^	E2	15.6	1.59
			173.67	0_2_,2^+^	E2	1.00	1.00
1496.45	0.03 < *τ* < 1.09	0_3_,2^+^	1496.45	0,0^+^	E2	1.00	1.00
			1403.26	0,2^+^	E0+(M1 and/or E2)	2.4^b^	1.43
1513.83	0.94 < *τ*	4,4^+^	1420.65	0,2^+^	E2	1.00	0
			1207.20	0,4^+^	E2(88±10 %)	3.55	0
			881.66	0,6^+^	E2	0.98	0
			339.30	2,2^+^	E2	1.00	1.00
			245.30	2,3^+^	E2	2.06	0.56
			129.37	2,4^+^	E2	4.65	0.20
1818.28	0.24 < *τ* < 1.04^c^	0_5_,2^+^	1725.13	0,2^+^	E0+(M1 and/or E2)	1.25^b^	0.56
			1511.74	0,4^+^	E2	1.00	1.00
			618.95	0_2_,0^+^	E2	1.00	1.00
			541.59	0_2_,2^+^	E2(50±11 %)	4.74	1.43

a The result of a previous measurement by Coulomb excitation [20,19] was 0.90±0.03 ps.

b No information is available on the E2 component of the transition; *B*(E2) value has been calculated as an upper limit assuming 100 % E2.

c The result of a previous measurement by Coulomb excitation [20,19] was (1.3±0.3) ps.

## References

[1] Bohr A, Mottelson B (1975). Nuclear Structure.

[2] Wu X (1994). Phys Rev C.

[3] Börner (1991). Phys Rev Lett.

[4] Oshima M (1995). Phys Rev C.

[5] Garrett PE (1997). Phys Rev Lett.

[6] Fahlander C (1996). Phys Lett B.

[7] Korten W (1993). Phys Lett B.

[8] Carminboeuf F (1997). Phys Rev C.

[9] Börner H, Jolie J (1993). J Phys G: Nucl Part Phys.

[10] Dewey MS (1989). Nucl Instrum Meth A.

[11] Kessler EG (1988). J Phys G: Nucl Phys.

[12] Haque AMI (1986). Nucl Phys A.

[13] Khoo TL, Løvhoiden G (1977). Phys Lett B.

[14] Sheline RK (1993). Pramana.

[15] Sheline RK (1993). Phys Rev C.

[16] Oothoudt MA, Hintz NM (1973). Nucl Phys A.

[17] Burke DG (1994). Nucl Phys A.

[18] Robinson SJ, Jolie J (1992). ILL Internal Report 92RO15T.

[19] Ronningen RM (1977). Phys Rev C.

[20] Browne E (1994). NDS.

[b21-j51deh] 21R. C. de Haan et al., (1999) to be published.

[22] Casten R (1990). Nuclear Structure from a Simple Perspective.

[23] Hager RS, Seltzer EC (1968). NDT.

